# OSA Wellness Scale (OWS): A New Health-Related Quality of Life Test in Obstructive Sleep Apnea Patients Treated with Mandibular Advancement Device

**DOI:** 10.1155/2022/4629341

**Published:** 2022-09-21

**Authors:** Domenico Ciavarella, Alessandra Campobasso, Laura Guida, Angela Pia Cazzolla, Graziano Montaruli, Fabrizio Corlianò, Luigi Romano, Michele Cassano, Michele Tepedino, Giuseppe Burlon

**Affiliations:** ^1^Department of Clinical and Experimental Medicine, University of Foggia, Clinica Odontoiatrica Via Rovelli 50, Foggia 71122, Italy; ^2^Department of Otolaryngology Head and Neck Surgery, San Bassiano Hospital, Via Dei Lotti 40, Bassano Del Grappa (VI) 36061, Italy; ^3^Department of Biotechnological and Applied Clinical Sciences, University of L'Aquila, V.le S.Salvatore, L'Aquila 67100, Italy

## Abstract

**Objectives:**

To present a new short self-test, called the OSA wellness scale (OWS), for assessing the health-related quality of life (HRQoL) changes in obstructive apnea syndrome (OSA) patients treated with mandibular advancement device (MAD).

**Methods:**

51 OSA patients (8 women and 43 men, mean age 52.3) treated with a fully customizable MAD device (Protrusor) were retrospectively enrolled. Each patient received a home sleep apnea testing (HSAT) at baseline (T0) and after three months of MAD treatment (T1). Two self-test evaluations, the Epworth sleepiness scale (ESS), and OWS were also submitted at T0 and T1. The OWS was a short self-test of 8 questions for evaluating the daytime HRQoL. Patients gave an assessment from 0 to 3 for each question. At the end of the questionnaire, the patients had a score from 0 to 24, resulting from the sum of all 8 scores. The higher the score, the greater the patient's perceived state of discomfort.

**Results:**

At T1, a significant decrease in the oxygen desaturation index (ODI) and apnea-hypopnea index (AHI) was shown (*p* < 0.0001), while no significant changes in body mass index (BMI) were found. Both the ESS and the OWS records showed a significant reduction in daytime sleepiness and HRQoL (*p* < 0.0001).

**Conclusion:**

The OWS could be a useful method to verify and numerically compare the perceived quality of life in OSA patients, before and after MAD therapy.

## 1. Introduction

Obstructive sleep apnea (OSA) is a respiratory disease characterized by repeated episodes of upper airway collapse and obstruction during sleep associated with arousal from sleep, with or without oxygen desaturation [1]. The obstruction may be complete (apnea) or partial (hypopnea) during sleep. It has long been recognized that men have greater vulnerability than women toward developing OSA [1]. The ratio of men to women is in the range from 5 to 8 : 1 because women are hormonally protected until menopause, after which the relationship tends to be equal [2]. The consequences of OSA are related to two important aspects: sleep time quality modification and respiratory gas exchange alteration [3]. The complete overnight laboratory polysomnographic evaluation (PSG) is required in severe cases and remains the gold standard for OSA evaluation [4]. Considering the continuous increase in sleep disorders, sleep laboratories and their diagnostic strategies are in high demand, and alternative methods are proposed in the literature for the screening and diagnosis of OSA, such as home sleep apnea testing (HSAT) [5]. The HSAT is a level III diagnostic tool, that can be performed in home settings without sleep technologists, representing a less expensive diagnostic option for clinical diagnosis of OSA. Continuous night arousals alter the physiologic sleep cycle, changing sleep quality, daytime life, and health-related quality of life (HRQoL) [6]. Nocturnal arousals also generate a disturbing unrefreshing sleep, that causes daytime sleepiness, head and neck pain, loss of concentration, anxiety or depression, weakness, and sleep bruxism [7, 8]. Untreated obstructive sleep apnea (OSA) requires medical intervention and is related to reduced work performance and work-related injuries. The economic burden associated with untreated OSA is significant [9]. The economic impact of untreated OSA patients is related to the limitation of work performance, and to the increased number of occupational injuries, motor vehicle crashes, and frequent hospitalizations for cardiovascular pathologies (strokes and heart attacks). OSA is comparable to other chronic diseases and is very difficult to describe exactly its economic effects, but is estimated that OSA patients cause a huge economic burden on society [10].

The impact of OSA is related to quality of life (QLI) modifications in undiagnosed or untreated patients [11]. The International Society for Quality of Life Research (ISOQOL) suggests that health-related quality of life (HRQoL) is the health aspect of quality of life that focuses on people's level of ability, daily functioning, and ability to experience a fulfilling life that includes the subjective perception of physical, social, and psychological functioning [12]. The physiologic parameters are clearly evaluated by HSAT or PSG, but the diagnosis of OSA is complete only if it has included the acquisition of data regarding the psychosocial aspect of the patient [13]. Various tools to measure HRQoL and self-evaluation of daytime sleepiness have been used in epidemiologic studies and clinical trials assessing OSA management [3]. The most used HRQoL tests are the sleep apnea quality of life index (SAQLI), The Medical Outcomes Study Short Form 36 Element Healthy Survey (SF-36), Maugeri obstructive sleep apnea syndrome questionnaire (MOSAS), Quebec sleep questionnaire (QSQ), obstructive sleep apnea patient-oriented severity index (OSAPOSI), functional outcomes of sleep questionnaire, the Pittsburgh sleep quality index (PSQI), and Epworth sleepiness scale (ESS) [14, 15].

In the present paper, the authors present a new short health quality life test, called the “OSA wellness scale (OWS),” to evaluate the HRQoL modifications in OSA patients treated with a mandibular advancement device (MAD).

## 2. Materials and Methods

51 patients (8 women and 43 men, mean age 52.3) with a diagnosis of OSA were retrospectively enrolled in the present study from patients treated at the Department of Orthodontics, University of Foggia, Italy, in chronological order from March 2017 to November 2020. All the procedures of this research protocol have adhered to the Declaration of Helsinki and have been approved by the Ethics Committee of the University of Foggia (Approval no. 43/CE/2019). Written consent was signed by each patient.

### 2.1. Criteria for Patients Selection

The inclusion criteria were as follows: age greater than 20 years old, body mass index (BMI) lower than 34 kg/m2, OSA diagnosis confirmed by nocturnal polysomnography, and night treatment with a fully customizable MAD-type device.

Exclusion criteria were as follows: smoking habit, neurological disorders, or previous cervical trauma.

### 2.2. Instrumental Evaluation

Each patient received a home sleep apnea testing (HSAT) at baseline (T0). A second HSAT was performed after three months of treatment (T1) with a fully customizable MAD device (Protrusor® Dr. Burlon) [16]. The data from the HSAT recordings were used for manual scoring according to the American Academy of Sleep Medicine (AASM) criteria from 2012 [16]. The extracted data are described in detail in Table 1.

### 2.3. Self-Test Evaluation

For each patient, two self-test evaluations were submitted Epworth sleepiness scale (ESS) and OWS. The ESS is a patient's self-evaluation about daytime sleepiness but no information about the HQRoL modifications can be shown [17]. The OWS is a new patient emotive self-evaluation about the daytime HQRoL. Both EES and OWS were done before MAD treatment and after three months of treatment.

### 2.4. Description of OWS

**Figure 1:**
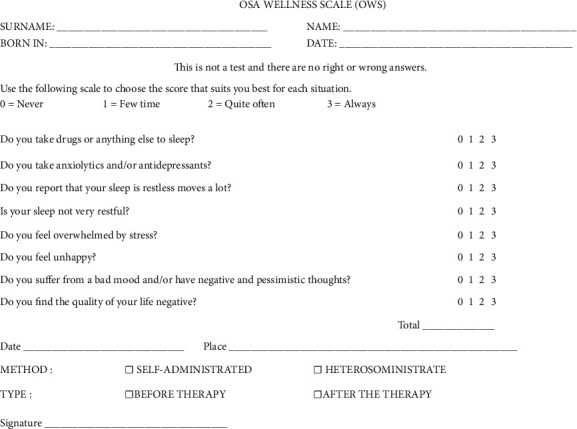
OSA wellness scale (OWS).

All patients were asked to complete the OWS questionnaire. The OWS is a short self-test to evaluate the daytime HRQoL subdivided into 8 questions (Figure 1). For each question, patients choose from 0 to 3 : 0 if the response is “never”, 1 if the response is “few times,” 2 if the response is “quite often,” and 3 if the response is “always”. At the end of the questionnaire, the patient has a score from 0 to 24, resulting from the sum of all 8 scores. The higher the score, the greater the patient's perceived state of discomfort.

Question 1 is used to evaluate the patient's drug assumption; question 2 evaluates the sleep quality time; the last 4 questions are used to evaluate the patient psychological aspect.

### 2.5. Statistical Analysis

Data were evaluated using Shapiro–Wilk normality test with a confidence level of 95%. A parametric Test (paired *t*-Test) was used for values that passed the normality test; a nonparametric (Wilcoxon Test) was used for values that unpassed the normality test (Tables 1 and 2).

## 3. Results

**Figure 2:**
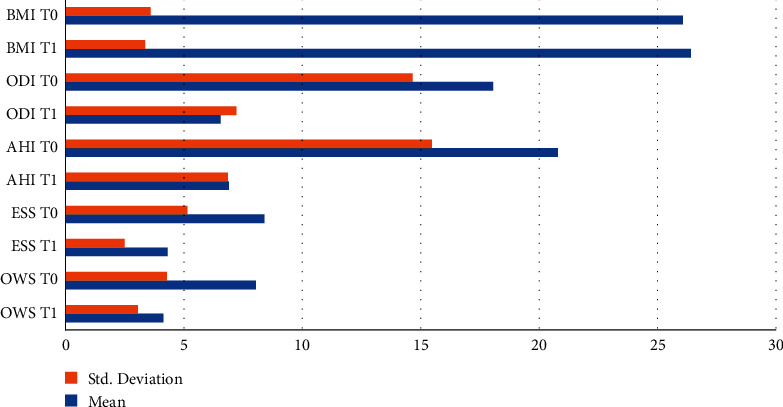
Bar chart of the evaluated records at T0 and T1.

Data and statistical evaluation were shown in Table 1. The mean difference of each evaluated parameters was shown in Table 2. Patients presented no significant modification of body mass index (BMI) after the MAD treatment (+0.3345; *p*=*n*.*s*.). A statistically significant modification was found in both the apnea-hypopnea index (AHI) and oxygen desaturation index (ODI) (*p* < 0.0001), which are the physiological parameters for assessing oxygen saturation. AHI decreased by −13.89 e/h (*p* < 0.0001); ODI decreased by −11.50 (*p* < 0.0001). After three months of treatment with MAD, the patients improved their daytime sleepiness and HRQoL. Both the ESS and the OWS records showed a significant reduction of −4,078 (*p* < 0.0001) and −3.902 (*p* < 0.0001) (Figure 2).

## 4. Discussion

In the present paper, the authors evaluated the changes in the physiologic oxygen parameters (i.e., AHI and ODI reduction) and the modifications of HRQoL, using a new simple self-test (OWS), for assessing the efficacy of MAD treatment in OSA patients. The MAD therapy is effective in patients who have an upper airway obstruction in sleep time prevalently in the supine position, or in patients who refuse the CPAP or upper airway surgery [1]. The forward and vertical movement of the mandible increases the upper airway area, improving the oxygen saturation (SO2) and inducing a reduction of AHI and ODI, recorded by HSAT or PSG [18]. The increase in oxygen saturation may reduce the risk of cardiovascular disease and stroke, as suggested for other OSA therapy such as CPAP or upper airway surgery [19]. A second main effect of upper airway modification is the reduction of night arousals. The reduction of night arousals has an influence on daytime sleepiness and quality of life [1]. Several types of research have actually focused on the importance of HRQoL. The HRQoL describes the somatic, mental and social well-being status [20]. The self-evaluation is the most used criterion to evaluate the social effect of the treatment [21]. The Medical Outcomes Study Short Form 36 Element Healthy Survey (SF-36) is one of the most used questionnaires showing reduced subjective health status and HRQoL, in patients with OSA [22]. The SF-36 is designed for use in clinical practice and research, health policy evaluations, and general population surveys. This questionnaire is widely used, consisting of 36 questions to evaluate the health condition and the relative burden of disease [23]. The score of the SF-36 has a range from 0 to 100: zero represents the worst and 100 represents the best HRQoL. A norm-based score of more or less than 50 represents a better or worse HRQoL respectively, compared to the average general population. The eight norm-based domains are united into one physical and one mental aggregated health scale [24]. In order not to discourage patients from answering so many questions and to acquire the answers more quickly, burden a shorter test has been used by many authors. A short form of SF-36 is the SF-12 questionnaire. The developers of the SF-36 have consequently suggested that a 12-item subset of the items may accurately reproduce the two summary component scores which can be derived from the SF-36 (the physical component summary score (PCS) and mental health component summary score (MCS)) [25]. Other self-report questionnaires have focused on psychological distress [26]. Many authors have emphasized a relation between nonphysiologic sleep and distress [27]. Psychological distress is defined as a state of emotional suffering characterized by symptoms of depression, anxiety, hyperarousal, and psychophysiological tension that may be expressed through somatic symptoms like insomnia, headaches, muscular pain, lack of energy, and exhaustion [28]. Psychological discomfort or psychiatric disorders are caused by sleep disorders both in the young and adult populations [29]. Standardized scales, such as the Maugeri sleep quality and distress inventory (MaSQuDI ± 17), measure and monitor sleep-related distress in patients with insomnia, obstructive sleep apnea syndrome (OSAS), central hypersomnia, and behavioral sleep disorders (BSD), a macrocategory that includes unusual nocturnal behaviors such as rapid eye movement (REM) behavior disorders, parasomnia, periodic limb disorders, restless legs syndrome, nocturnal eating disorders, and sleep-related eating disorders [28].

In the present paper, the authors have evaluated the changes in HRQoL using the new OWS test, in OSA patients treated with MAD. This type of test has 8 questions to assess the HRQoL and distress, before and after treatment. The OWS test is simple and focuses on the real modifications of the HRQoL and distress. The OWS test also gives information about the drugs taken by the patient. This aspect is very important to evaluate the initial psychological discomfort of the patient before starting with every type of OSA treatment (both CPAP and MAD). The most widely used self-tests do not give this type of information. A recent study found that 40 mg of controlled-release oral morphine did not worsen OSA in men, challenging traditional thinking that OSA is worsened by opioids. However, a positive effect on OSA is associated with a direct effect on central nervous system respiratory depression. So, clinicians should be cautious in giving excess opiates and sedatives, given the increased risks [30].

In the present paper, the authors have shown lower scores in OWS questionnaires completed by the same patients after treatment. The improvement of functional parameters improves happiness, the HRQoL, and reduces daytime sleepiness. The OSA wellness scale (OWS) is a practical, quick and inexpensive tool that is easy to understand and is aimed at all walks of life. It may be used by the clinicians for the initial screening or for the final post-treatment check. In addition to the Epworth scale, anamnesis, and polysomnography (or cardio-respiratory monitoring), this test could provide a broader view of the impact of OSA pathology on the state of the general malaise of the apneic patient; and thus, could help the clinician not exclude from therapy an OSA patient with low apnea/hypopnea (AHI) who psychologically perceives the same discomfort as an OSA patient with high apnea/hypopnea (AHI). The title “OSA wellness scale (OWS)” is deliberately presented in a positive form to avoid altering the patient's judgment in advance, predisposing him negatively. The questionnaire takes into consideration various situations of daily life, including the possible intake of drugs, and, for each of them, the subject must establish a vote whose sum quantifies the degree of discomfort of the subject himself. It is an 8-item test that can be self-administered or hetero-administered. In the presented study, all clinical cases are self-administered to avoid external alterations. Each item is rated on a scale from 0 (“never”) to 3 (“always”). The sum of the answers given in numerical form gives the final score, which can range from 0 to 24. The higher the score, the greater the patient's perceived state of discomfort. In other words, the lower the overall score, the lower the state of discomfort perceived by the patient, so a low score is a sign of improvement in quality of life-related to health.

## 5. Conclusion

The OSA wellness scale (OWS) should be considered as a method to verify and numerically compare the patients' perception of the quality of life, both before and after therapy, in OSA patients treated with MAD.

Further studies should be suggested to create a larger sample for the validation of the test.

## Figures and Tables

**Table 1:** Data and statistical analysis of the extracted records.

	BMI and TO	BMI and T1	ODI TO	ODI and T1	AHI and T0	AHI and T1	ESS and T0	ESS and T1	OWS and T0	OWS and T1
Number of values	51	51	51	51	51	51	51	51	51	51
Mean	26.07	26.40	18.05	6.547	20.79	6.900	8.392	4.314	8.039	4.137
Std. Deviation	3.594	3.359	14.66	7.211	15.47	6.859	5.142	2.494	4.280	3.060
Std. Error of mean	0.5033	0.4703	2.053	1.010	2.166	0.9605	0.7201	0.3492	0.5993	0.4284
Lower 95% CI of mean	25.06	25.46	13.92	4.519	16.44	4.971	6.946	3.612	6.835	3.277
Upper 95% CI of mean	27.08	27.35	22.17	8.575	25.14	8.829	9.838	5.015	9.243	4.998
Normality test	Yes	Yes	No	No	No	No	No	Yes	No	No
*p*	ns		<0.0001		<0.0001		<0.0001		<0.0001	

BMI = body mass index; ODI = oxygen desaturation index; AHI = apnea-hypopnea index (AHI); ESS = Epworth sleepiness scale; OWS = OSA wellness scale.

**Table 2:** The mean difference of each evaluated parameter.

	BMI T0 and T1	ODI T0 and T1	AHI T0 and T1	ESS T0 and T1	OWS T0 and T1
Number of values	51	51	51	51	51
Mean	0.3345	−11.50	−13.89	−4.078	−3.902
Std. Deviation	1.366	12.76	13.35	4.408	3.874
Std. Error of mean	0.1913	1.787	1.869	0.6173	0.5425
Passed normality test (alpha = 0.05)?	No	No	No	No	No

BMI = body mass index; ODI = oxygen desaturation index; AHI = apnea-hypopnea index (AHI); ESS = Epworth sleepiness scale; OWS = OSA wellness scale.

## Data Availability

The individual data used to support the findings of this study are available from the corresponding author upon request.
